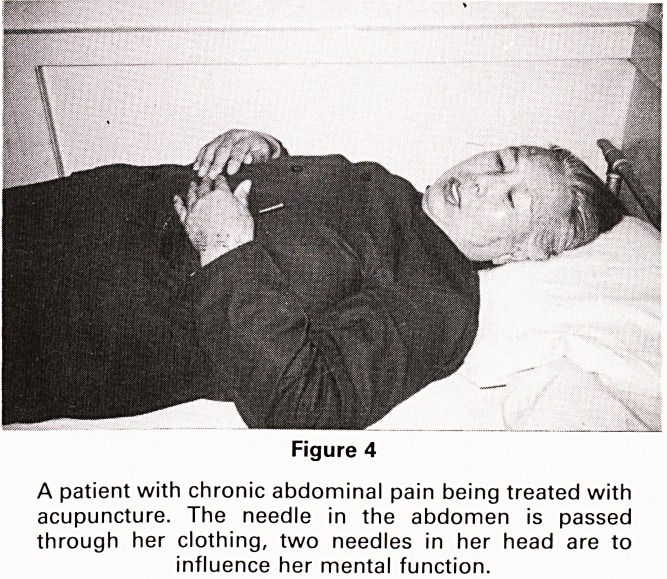# From Our Foreign Correspondent

**Published:** 1986-10

**Authors:** Andrew Williams, Erica Jones

**Affiliations:** Department of Medicine Bristol Royal Infirmary; Barrow Hospital


### From Our Foreign Correspondent

### Health Care in China today

We have recently returned from the People's Republic of China after completing a health tour of four cities (Peking, Nanking, Jinan and Shijiazhuang), and the neighbouring countryside as the guests of the All China Youth Federation.

We visited hospitals, communes, factories and kintergartens, and had the opportunity to talk to many health care professionals. We were overwhelmed by the warmth of our reception, and the friendship shown to us by the Chinese people.

Since the revolution in 1949 health care in China has undergone radical reform, western-style medicine has become firmly established alongside the traditional Chinese medicine, and is the first choice for the majority of young Chinese, particularly in the urban areas. These changes have become particularly noticeable since the ending of the cultural revolution in 1976, and the adoption of a pro-West stance by China.

The general practitioner does not exist as such in the Chinese medical system. Primary health care is based upon your place of work either in the factory or the agricultural commune. These have their own clinics with separate departments of medicine (western), surgery, obstetrics and gynaecology, pediatrics, dentistry, and traditional Chinese medicine. The facilities provided were always adequate and often good compared with western expectations. The larger factories and communes often had their own hospitals with inpatient facilities and operating theatres. Well-person check ups were the norm at annual and biannual intervals among the work force. To our surprise health care was not free in China. The patient paid, but he was reimbursed by his employer.

China has an active health education and family planning programme as witnessed by many large health posters seen throughout the country. The Chinese are encouraged to marry late (in their late 20's) and have only one child. Financial penalties are imposed on those with more than one child, and the posters depict the young couple with one child as being happier and healthier! (Fig. 1).

**Figure 1**

Health care poster—shows you will be wealthier, happier and more prosperous with just one child.

**Figure 2**

Shows a child with split bottom trousers, popular throughout China. The split was unfastened and left open, the child wearing no underpants. Whenever the need to defaecate was experienced the child would proceed without hindrance even in the most public of places.

All women in China work, and every factory, hospital and commune has its own kintergarten for the children of its employees. Pregnant women work until their delivery and are then allowed one month's maternity leave. Upon return to work they are allowed one hour for breast feeding each day.

The Chinese are encouraged to look after their aging parents, and grandparents are expected to mind their grandchildren when the parents are at work. Old people's homes do exist for those who have no living dependents, but they are not common. This however might change as the Chinese live longer with the higher standard of health care they are enjoying combined with the reduced number of children they are having. The home we visited was attached to a rural commune. The average age of the occupants was 74 years, and in contrast to Britain there were more men than women. They lived in bright, clean stimulating surroundings and were obviously happy and well cared for.

We visited provincial hospitals in Nanking, Jinan and Shijiazhuang.

These were hospitals that practiced western-style medicine although they all had Departments of traditional Chinese medicine which involved the use of acupuncture and moxibustion and traditional herbal medicines. (Figs. 3 and 4).

Acupuncture and moxibustion are two distinct therapeutic modes frequently used in conjunction in traditional Chinese medicine. Acupuncture treats disease by puncturing certain points of the human body with metal needles. Moxibustion is the application of heat produced by igniting moxa-wool and placing it on the skin. Moxa wool consists of dry mugwort leaves (Artemisia vulgaris). Mugwort grows throughout China and has been used for curative purposes for several thousand years. The heat generated from burning moxa-wool is mild and well tolerated but penetrated deep beneath the skin.

Both acupuncture and moxibustion promote the circulation of blood and adjust 'qi' (life force) within the 'channels' which link the zang-fu organs (heart, liver, spleen, lung, kidney) the fu organs (stomach, small and large intestine), the sanjiao (skin), brain and uterus.

The medicine practiced was similar to our own with

**Figure 3**

Moxibustion being used to treat a child with enuresis.

**Figure 4**

A patient with chronic abdominal pain being treated with acupuncture. The needle in the abdomen is passed through her clothing, two needles in her head are to influence her mental function.

some exceptions. Hyperbaric oxygen treatment was very popular and two hospitals visited had very large hyperbaric oxygen chambers. They were used to treat a variety of conditions including stroke. Often patients would receive acupuncture and moxibustion in combination with 'western' drugs. We saw acupuncture being used to treat hypertension, enuresis, chronic abdominal pain and aid stroke recovery. Acupuncture was not used to provide anaesthesia in surgery except for operations on the head and neck.

A two tier health service is practiced in China in that high ranking officials in the communist party are eligible for treatment in separate better equiped outpatient and in-patient suites in the provincial hospitals. Costs charged were higher, but wealth alone could not give you access to these facilities.

All the places visited were memorable, but three deserve special attention. We were the first British group to visit Shandong Psychiatric Hospital in Jinan. All the hospital doctors were present to welcome us. The hospital was built in 1956, had 350 beds, 402 medical workers including 37 doctors. The average length of stay in this hospital was 2 to 3 months, and the majority were admitted with an acute psychoses (schizophrenia or mania). Depression was rarely a cause of hospitalisation. Alcoholism and drug abuse are rare, as are overdoses and suicide. Treatment programmes are similar to ours with the use of neuroleptics and E.C.T., but insulin coma therapy is still practised, and acupuncture is used in anxiety states and insomnia. The Chinese have a longer tradition in psychotherapy than westerncultures and indeed in this hospital both individual and group therapy were standard practice. A Mental Health Act does not exist in China, and if a patient refused to come into hospital, he will be visited in the community by a psychiatrist or psychiatric nurse, and maintained there if possible (Family sick bay) or if not he will be forced into hospital by his family.

We visited the Norman Bethune Memorial Hospital in Shijiazhuang. This is a military hospital and is named after a Canadian surgeon who came and worked in China during the communist revolution and helped the Chinese resist the Japanese invasion. He so impressed the Chinese with his work, and self-sacrificing manner that after his premature death from septicaemia following a scalpel injury whilst operating, a hospital was named in his memory and his statue stands in the entrance.

Hebei medical college inShijiazhuang was visited during its celebration of its 70th birthday. The reception we received from the students and staff was overwhelming and we made many friends there. The medical school has 2,800 students and 600 members of staff. The course is similar to ours with a three year clinical period, and a two year preclinical one. All the students learn medical English and also have a course in traditional Chinese medicine. English is the most popular foreign language learned in China and all the medical students were keen to practice theirs. A nationwide television programme teaching English has an enormous following, and large crowds collect outside the foreign languages book store in Peking on a Sunday morning to practice their English.

The cultural revolution (1966–1976) was a disruptive time in China particularly for the Universities and medical schools. The universities were closed, no students were admitted and the University Directors and other prominent academics were forced to work as peasants on the land or in factories (stoking a boiler in one particular case). As a consequence no doctors were trained, and patients were not treated as well as they might have been. During this period the barefoot doctor was created, a paramedical person with limited medical training who worked in the countryside. Apart from in remote rural areas the barefoot doctor is now a feature of the past.

The Chinese government since 1949 has radically improved the social conditions and basic health care of the ordinary Chinese. The current leadership realises that future progress will be facilitated by greater friendship and co-operation with the west.

We were impressed by the health care we saw in China, and by the friendliness and frankness of the Chinese we met. In particular we would like to thank the All China Youth Federation and Gordon Burnett of Interchange, London for organising the trip, and we hope that more health exchanges between our two countries will be possible in the future.

Andrew Williams, BSc, MRCP
Department of Medicine
Bristol Royal Infirmary
Erica Jones, MB, ChB(Hons), MRCP
Barrow Hospital

## Figures and Tables

**Figure 1 f1:**
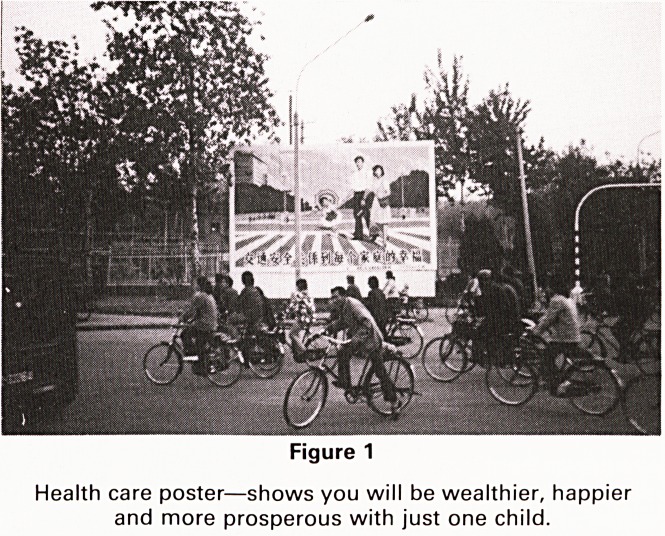


**Figure 2 f2:**
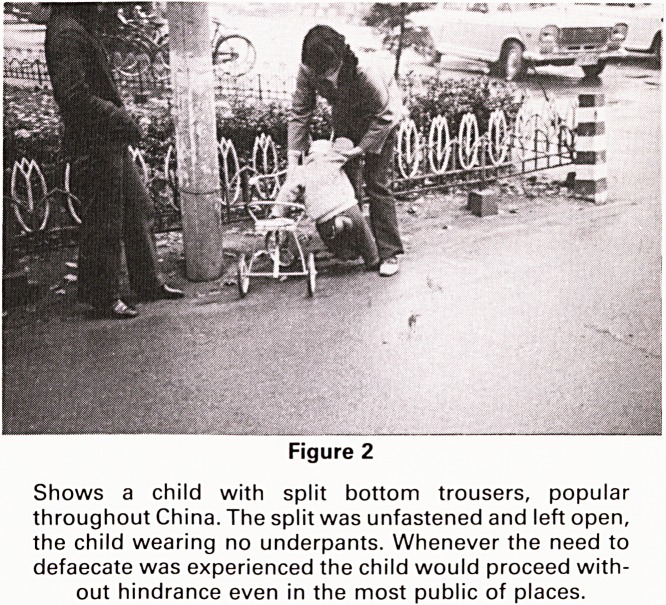


**Figure 3 f3:**
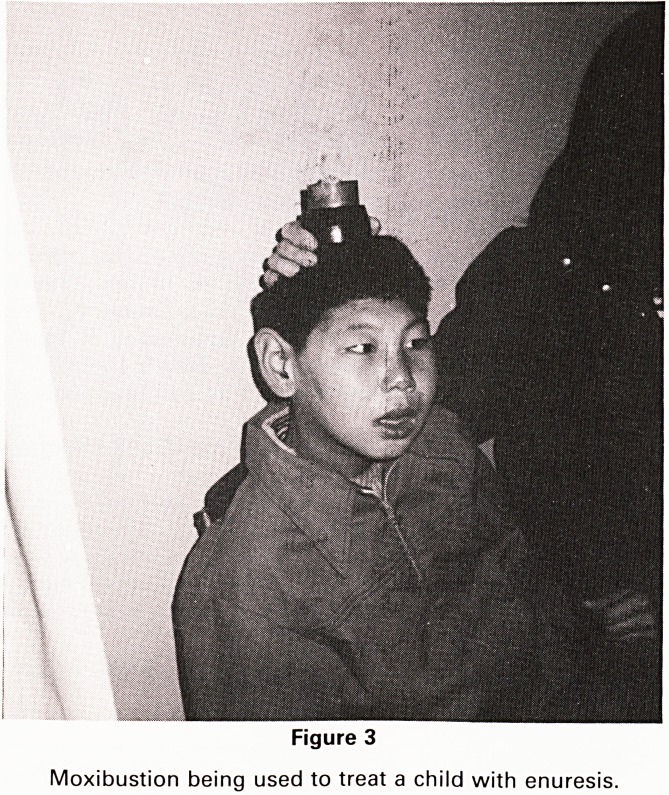


**Figure 4 f4:**